# Mycobacteriophage CRB2 defines a new subcluster in mycobacteriophage classification

**DOI:** 10.1371/journal.pone.0212365

**Published:** 2019-02-27

**Authors:** Cristian Alejandro Suarez, Jorgelina Judith Franceschelli, Héctor Ricardo Morbidoni

**Affiliations:** Laboratorio de Microbiología Molecular, Facultad de Ciencias Médicas, Universidad Nacional de Rosario, Rosario, Argentina; University of York, UNITED KINGDOM

## Abstract

Mycobacteriophages are viruses -mostly temperates- that infect *Mycobacterium smegmatis* and sometimes *Mycobacterium tuberculosis*. Mycobacteriophages are grouped in clusters on the basis of the overall nucleotide sequence homology, being further divided in subclusters as more mycobacteriophage genomes are sequenced and annotated. As part of our on-going screening for novel isolates, we herein report the bioinformatics analysis of CRB2, a mycobacteriophage belonging into the *Siphoviridae* family that propagates at 30°C. CRB2 has a 72,217 bp genome with a 69.78% GC content that belongs to Cluster B; nucleotide comparison with other B cluster members positions CRB2 as the sole member of a new subcluster, B9, being mycobacteriophage Saguaro (belonging into subcluster B7) its closest relative. Sequencing and annotation of 14 mycobacteriophages isolated by our group has yielded six cluster A members, a singleton, four of the five members of subcluster B6, one of the three reported members of subcluster G4, and CRB2 which defines subcluster B9. Considering the massive mycobacteriophage search performed in USA and the relatively rarity of our phages, we propose that factors other than size of the sampling determine the variability of mycobacteriophage distribution, and thus a world-wide concerted mining would most likely bring extremely rare and yet undiscovered mycobacteriophages.

## Introduction

Bacteriophages are bacterial viruses that specifically infect bacteria; the infection process leads to two types of general outcomes, lysis of the bacterial hosts or establishment of the bacteriophage either as a prophage (thus integrating into the bacterial chromosome) or partitioning as a plasmid. Bacteriophages are the most abundant and diverse biological forms on earth, and has been used for decades as a simple yet highly valuable tool to explore bacterial genetics. Currently, bacteriophages capable of infecting members of the genus *Mycobacterium* (dubbed mycobacteriophages) constitute the most studied group with almost 10,000 phages isolated so far, of which the vast majority (9,700) were obtained by using the saprophytic fast-growing mycobacterial species *Mycobacterium smegmatis* as host; opposite to that only a handful of phages have been isolated using other mycobacterial species including the pathogen *Mycobacterium tuberculosis* [1,2]. Through a joint effort between Howard Hughes Medical Institute, several universities and colleges and (in a very fresh approach) with the participation of students from numerous high schools (mostly U.S. based), Graham Hatfull and his group (Pittsburgh Bacteriophage Institute and Department of Biological Sciences, University of Pittsburgh) led the work leading to the sequencing and analysis of a very large number of genomes (1643) of the above mentioned phages, creating a massive amount of information from which the processes underlying mycobacteriophage genomic evolution started to become clear [3–5]. A major factor in the evolution of mycobacteriophages is due to the illegitimate recombination that produces a mosaicism of the genome sequences, creating countless variations [3]. Hatfull´s group used the mass information not only to lay down the foundations for the classification and grouping of mycobacteriophages but also for developing genetic tools for mycobacterial manipulation [6]. Moreover, those researchers extracted very interesting pieces of information of the analysis of each group; i.e. the finding of the mechanism of exclusion of mycobacteriophages by phage Fruitloop interacting with Wag31, a mycobacterial protein involved in cell division [7]. We have conducted a much more reduced size search looking for mycobacteriophages locally, using *M*. *smegmatis* as host and 30°C as the temperature for direct isolation of mycobacteriophages [8,9]. The analysis of one of such mycobacteriophages, named CRB2, defining a new subcluster, is reported herein. Our results show that CRB2 is a lytic non-transducing mycobacteriophage, member of the *Siphoviridae* family; bioinformatics analysis of its genome sequence allowed establishing it as the sole member of a new subcluster (designated B9), being mycobacteriophage Saguaro (subcluster B7) the most closely related to it up to this date.

## Material and methods

### Culture media and chemicals

Middlebrook 7H9 broth additioned of 10% ADS (albumin-dextrose-NaCl) supplement, 0.5% (w/v) glycerol (hereafter designated as 7H9-ADS-Gly for short) and 0.2% (w/v) Tween 80 was used for liquid growth experiments. Solid medium for strain propagation was Middlebrook 7H9-ADS-Gly with the addition of 1.5% (w/v) agar. All chemicals, enzymes and solvents were from Sigma- Aldrich (Mo) unless stated differently.

### Isolation and characterization of mycobacteriophages

The protocols for direct isolation (that is without an enrichment step) and propagation of mycobacteriophage CRB2 have been described elsewhere in detail [9]. Briefly, environmental samples (mostly soil) from different regions of Argentina, where resuspended in Phage Buffer (PhB, 50 mM Tris-HCl pH 7.6, 150 mM NaCl, 2 mM CaCl_2_, 10 mM MgS0_4_) and gently stirred overnight, centrifuged and filter sterilized. The presence of phages in those filtered samples was detected by adsorption of 100–500 μl of filtrate to 100 μl of late log fresh culture of *M*. *smegmatis* mc^2^155 for 30 min at room temperature, followed by addition of 4 ml of molten warm top agar, mixed gently and poured on top of 7H9-Gly-Ca agar plates. After incubation for 24–48 h at 30°C or 37°C, plaque size, morphology and turbid/clear appearance were scored, single lysis plaques were picked and resuspended in PhB to release the phages. Further amplification generated a stock lysate which was kept at 4°C.

### CRB2-mediated transduction assays

The ability of CRB2 to transduce chromosomal genes was assessed by two protocols, in first place a *M*. *smegmatis* mc^2^155 strain in which a gene involved in biotin biosynthesis was deleted and replaced by a Kanamycin marker [9] (thus Δ*bio*C Km^R^) was used as donor strain. CRB2 was propagated in this strain according to standard protocols used for the transducing mycobacteriophage Bxz1 [10] and subsequently used to infect the parental *M*. *smegmatis* mc^2^155 strain selecting for Km resistance. The clones obtained in that process were screened for biotin auxotrophy. A second protocol used -reported by Sander and Schmieger [11]—consisted in treating the lysate produced by CRB2 on *M*. *smegmatis* mc^2^155 with DNAse, followed by PCR amplification of genes MSMEG_1886 and MSMEG_4724 (chosen as they were being studied at this lab); for this purpose suitable diagnostic primers were used as follows: MSMEG_4724-Fw, GAATTCCGGTTTGGCCAGCCCGGACAG; MSMEG_4724-Rev, CTTAAGGAGTCGCGCGGTGTCGACTC; MSMEG_1886-Fw, TCTAGATGGAAGGCCGCCGGTGTGGGCGAG; MSMEG_1886-Rev, GAATTCGAGAACCAGGCGTACCTAGGCGGC. Briefly, for DNA extraction from mycobacteriophage virions, 4ml of high titer -10^10^ Plaque forming Units (PFU)/mL- lysates generated as previously described [9] were centrifuged 1 h at 14000 rpm; the supernatant was discarded and the pellet resuspended with gentle agitation at room temperature for 2 h in 600 μl of 10mM Tris HCl 1mM EDTA pH 8.0 (TE) Buffer; afterwards 50 μg/ml of DNasaI was added and the mixture incubated for 2 h at 37ºC in order to degrade contaminating *M*. *smegmatis* chromosomal DNA. After that step, SDS (15mg/mL) and Proteinase K (20μg/ml) were added followed by incubation at 56°C for 2 h. Finally, mycobacteriophage DNA was purified by phenol/chloroform and chloroform: isoamyl alcohol (24:1 v/v) extractions followed by DNA precipitation and collection according to Sander *et al* [11]. The DNA obtained was kept at -80°C until it was used. PCR amplification reactions contained 2.5 μl DMSO, 1.0 mM dNTPs, 30 pM of each primer, 1.5 mM MgCl_2_, 5 μl Taq buffer and 1 unit Taq Polymerase. Amplifications were carried by 25 cycles of denaturation (95°C, 30 sec), hybridization (58°C, 30 sec) and elongation (72°C, 90 sec). The samples were analyzed by standard agarose gel electrophoresis. In all cases mycobacteriophage Bxz1 [10] was used as control.

### Genomic sequencing

The genome sequencing of CRB2 was carried out at a commercial local facility (INDEAR, Instituto de Agro-Biotecnología de Rosario, Rosario, Argentina) using Illumina HiSeq 1500 technology. Libraries were generated by using the Nextera1XT DNA Sample Preparation Guide Illumina (October 2012, Illumina Inc, San Diego, CA, USA). To obtain the template bacteriophage DNA high-titer bacteriophage lysates (generated on plates using 0.4% (w/v) agarose top medium) were treated as described previously with DNAse and RNAse treatment and filtration through 0.2 μm cellulose acetate filters followed by virion disruption by the addition of guanidine thiocyanate (Sigma, final concentration 800 mg/mL). After this step, was added to the cleared lysates and the mixture was gently mixed at room temperature for 2 h for full solubilization of this salt. Bacteriophage DNA was extracted from this suspension by using Wizard DNA Clean-Up System (Promega) according to the manufacturer instructions. Quantitation of bacteriophage DNA was estimated at Abs_260_ nm and DNA integrity was assessed by agarose gel electrophoresis. DNA was finally stored at -20°C until further use.

### Bioinformatics analysis

Genome annotation of open reading frames was accomplished by using GeneMark and Glimmer included in the DNAMaster program package (http://phagesdb.org/DNAMaster/). The presence of tRNA was analyzed by Aragorn and tRNA Scan SE (available at http://cobamide2.bio.pitt.edu). The probable functions of encoded proteins were obtained by using HHPred [12] and HMMER [13]. Genomic parameters (ORF density, gene length, coding percentage and GC% content) were obtained from Artemis version 16.0.0 [14]. Alignment and phylogenetic network were performed with Gegenees 2.2.1 using a 50 bp fragment-size and a 25 bp step-size, nexus file generated was analyzed by Splitstree software using NeighborNet method [15,16]. The average nucleotide identity (ANI) was calculated using DNAMaster genome comparison tool. Afterwards, the ANI heatmap was constructed in R version 3.4.4 using heatmap.2 included in gplots 3.01 package (https://cran.r-project.org/web/packages/gplots/index.html). A total of 159 mycobacteriophage genomic sequences belonging to cluster B were obtained from NCBI and Actinobacteriophage databases (http://phagesdb.org/) as shown in S1 Table. If necessary, the Genbank files were generated and edited in DNAMaster for the inclusion in the Phamerator database [17]. Genome distance was evaluated using Mash software available in Github (https://github.com/marbl/mash). For this analysis, we randomly chose a total of 74 genome sequences of mycobacteriophages belonging to different subclusters B (1 to 8) and CRB2. The parameters used were sketch size of 50000 and *k*-mer size of 17 [18,19]. The Mash distance was represented as a dendrogram by using dendextend and circlize libraries [20,21] and average method in hclust package in R. Alignment and phylogenetic studies were performed by Gegenees, ClustalW and MEGA7 [22]. Jalview 2.10.5 was used to visualize and edit the ClustalW alignment [23].

Phage Tape Measure Protein (TMP) sequences were obtained from GenBank or from http://phagedb.org/ database. Gepard [24] was used to generate the dotplot of TMP protein sequences from 75 mycobacteriophages belonging to different B subclusters included in the 160 mycobacteriophage set used throughout this study.

The multivariate data analysis was performed in R using FactoMineR package. For the principal component analysis (PCA) the %GC content and genome size of the mycobacteriophages were obtained from http://phagedb.org.

The alignment-free program CAFE [25] was used to study the relationship between the 75 mycobacteriophage set. The dissimilarity distance was performed by using Manhattan, a *k*-mer counts method with a lenght of *k* = 8. The dissimilarity matrix obtained was used to generate a dendrogram using UPGMA algorithm in R.

Gene content dissimilarity (GCD) for each mycobacteriophage was determined using Python scripts developed by Mavrich and Hatfull available at https://github.com/tmavrich/mavrich_hatfull_nature_micro_2017 [19]. The data for the scripts were obtained from the Phamerator local database of the 160 mycobacteriophages. The boxplot graphic was made in R.

The relative synonymous codon usage (RSCU) analysis was accomplished as described by Esposito *et al*. [26]. Briefly, coding features were obtained from NCBI of each 75 mycobacteriophage analyzed except for CRB2. MEGA 7.0 was used to calculate the RSCU values for each genome. The clustering analysis was performed by comparing the RSCU values between all mycobacteriophages genomes using Pearson´s correlation coefficient that was calculated in R. The distance matrix was constructed using the equation d = (1—r) x 100, where d is distance and r is the Pearson coefficient. The UPGMA dendrogram was created in R software.

### Nucleotide accession number

The nucleotide sequence of CRB2 has been deposited in GenBank (Accession Number MK059749).

## Results and discussion

### CRB2 is a lytic non-transducing mycobacteriophage

Our initial efforts, started in 2007, were directed to the isolation and subsequent study of mycobacteriophages isolated from Argentina, being that the first study undertaken in South América [9]. Since the extensive work by Hatfull´s group was carried out looking for mycobacteriophages at 37°C, we decided to look for mycobacteriophages capable of replicating at 30°C. Our strategy–with no enrichment step- granted us several mycobacteriophages, of which surprisingly, roughly 60% were characterized for a restricted temperature of replication in *M*. *smegmatis*, being able to propagate at 30°C but not at 37°C [9]. As part of the completion of the sequencing project one of those phages -designated as CRB2- was further characterized and found to propagate at 30°C, yielding plaques of 1–1.5 mm in diameter, albeit with a turbid appearance. Importantly, no stable lysogens were obtained from several plaques (data not shown). Also we previously determined by Pulse Field Gel Electrophoresis (PFGE) that CRB2 generates chromosomes of somewhat variable length, so it most likely has redundant ends and packages its DNA by a headful-packaging system [9]. Bacteriophages that use this packaging strategy can generate virion particles containing host DNA [27]. Chromosomal gene transduction mediated by CRB2 proved to be unsuccessful in spite of testing two different protocols, however Bxz1, a well-characterized transducing mycobacteriophage used as control, yielded biotin auxotroph transductants with a frequency of 10^−7^, mycobacterial genes were also detected by PCR amplification in DNA extracted from DNAse treated Bxz1 lysates but not form lysates produced by CRB2 (data not shown). Importantly, there are only two reports of transducing mycobacteriophages, I3 and Bxz1 [10,28], in spite of more than 1,600 mycobacteriophages isolated so far.

### Genomic analysis of CRB2

The phage DNA genomic sequence was assembled and analyzed through the use of several bioinformatics tools. Artemis showed a 69.78%GC content along its 72,217 bp length; and a 95% coding percentage represented in 96 ORFs with a gene density of 1,3 gene/kbp. Glimmer and GeneMark in DNAMaster were used for protein-coding gene prediction; 38 coding sequences were detected in the leftward transcribed strand and 58 in the rightward transcribed strand. BLASTP and HHpred predicted the function of 1/3 of the ORFs (29 ORFs) while almost 2/3 (67 ORFs) were hypothetical coding sequences and 2 had no homology to any protein available in public databases. The search for tRNA genes using Aragorn showed their absence in the CRB2 genome. Comparison using BLASTN with the Actinobacteriophage database (available at http://phagedb.org, last accessed 09/30/2018) with other mycobacteriophage genomic sequences showed high nucleotide sequence similarity with phages in cluster B. Phage Saguaro belonging to subcluster B7 was the most similar to CRB2.

### CRB2 genome annotation and organization

As mentioned above, the CRB2 genome revealed 1/3 of its predicted ORFs having a probable function; remarkably, most of them presented higher homology in BLASTP analysis to proteins of phage Saguaro (49/96), the only member of subcluster B7 (S2 Table). A similar finding was observed when comparing proteins of unknown function or hypothetical proteins with a total of 27 ORFs displaying high homology to Saguaro´s counterparts. Importantly, most of the remaining ORFs (some with predicted function but most of them having unknown function or being hypothetical proteins) showed good homology to proteins of phages from cluster B, especially those belonging into subcluster B4 (11 ORFs) followed by phages of the subcluster B6 (KayaCho, Jolie1, Hosp, 39HC and 40BC) with a total of 9 ORFs. Also of importance is the fact that 4 ORFs (30, 54, 77 and 85) were homologous to ORFs present in the genome of phage Thonko, a recently isolated phage that defines a new subcluster, B8, according to the information available at http://phagedb.org.

Taken together, although a minor fraction of the total proteins are also present in phages of B subclusters as well as of other clusters and, the vast majority of CRB2 ORFs are homologous to ORFs present in subclusters B4, B6, B7 and B8. Since both B6 includes only five phages while B7 and B8 are represented by only one phage each, we anticipated that CRB2 would be highly related to those phages.

**Fig 1:**
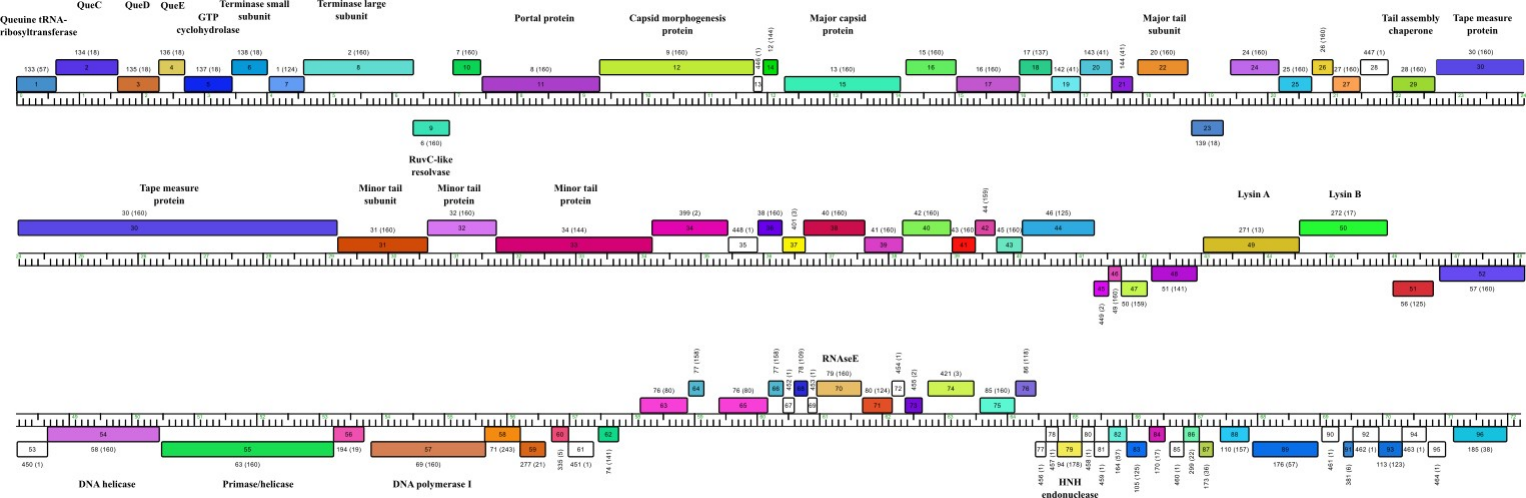
Genome map of mycobacteriophage CRB2. The map was generated using Phamerator [17]. Genes are shown as colored boxes, according to pham assignments for each gene (gene members of a phamily have the same color); gene names are shown within the boxes, with each pham number of that gene shown above with the number of pham members in parentheses. Genes shown in white are orphans and have no mycobacteriophage relatives in our database. Treshold values for considering genes relates are: greater than 32.5% amino acid identity or a BLAST E value lower than 1e-50. Identified gene functions are displayed in bold. Genes depicted above or below the genome are transcribed rightwards or leftwards respectively.

A subset of 160 fully sequences genomes of mycobacteriophages of cluster B (S1 Table) that were freely available in public databases, along with CRB2, were includedin a Phamerator local database The sequence of phage Saguaro was initially obtained from Actinobacteriophage DB (phagesdb.org) and at later stages of this work was also available from NCBI. The phams assignation obtained through Phamerator using our local database showed that CRB2 contained 17 ORFs that were not present in our local database of 160 mycobacteriophages, with other 4 ORFs that belong to *phams* only described for phage Saguaro. Genome organization was comparable to that of members of the *Siphoviridae* family; with a syntenic group of genes encoding DNA packaging, virion structural and assembly proteins (Fig 1).

The ends of the genome of CRB2 are circularly permuted genome as we have described previously [9], thus in order to align and compare its genome to other mycobacteriophages, the left end of CRB2 is arbitrarily positioned at the first noncoding region at the left of the terminase large subunit. Interestingly, the first six genes (1–6) located at the left end of CRB2 have strong sequence similarity to the first six genes encoded by Saguaro and the subcluster B2 phages. ORFs 3–5 displayed homology to *queC*, *queD* and *queE* genes, which are known to encode enzymes carrying out the biosynthesis of preQ_0_, a precursor of queosine (Q) which is a 7-deazaguanine derived from GTP that is found in eukaryotic and bacterial tRNAs. Queosine incorporation into DNA could cause resistance to several host endonucleases [29]. The enzyme queuine tRNA-ribosyltransferase encoded by ORF2 is a DpdA-like protein, and thus, is possibly involved in the insertion of preQ_0_ into DNA. DpdB (a member of the ParB superfamily) is generally present whenever DpdA is present therefore suggesting that ORF1 could encode a *dpdB* homolog [29]. The description of those genes in CRB2 matches findings described by Hatfull´s group showing that, in spite of the lack of evidence for the presence of queuosine as a tRNA modification and /or the known queosine biosynthetic genes from mycobacterial genomes, this modification is a feature of several B subcluster phages.

The DNA packaging module is composed of two side by side genes (ORF7 and ORF8) that encode the small and large terminase subunits respectively, and the portal protein (ORF10). Upstream of the terminase large subunit gene, ORF9 encodes a protein homologous to RuvC, as has also been reported for phages of subclusters B1, B2 and B4. The function of this protein may be related to virion assembly through resolution of the Holliday junction [30]. From ORF12 to 33 we found structural and virion assembly genes intercalated with hypothetical proteins. ORF30 encodes the TMP which is 2160 AA long, similar to that of phage Saguaro (2183 AA), both are among the longest TMP present in phages of cluster B.

The DNA replication module consists of three genes, a DNA helicase (ORF54), a putative primase/helicase (ORF55) and a DNA polymerase I (ORF57); meanwhile ORF56 displays homology to hypothetical proteins present in other mycobacteriophages such as phage Godines (S2 Table). Lastly ORF49 and ORF50 encode the components of the lysis cassette; the first one encodes a protein with a N-acetylmuramoyl-L-alanine amidase (E.C. 3.5.1.28) domain, while HMMER predicted two different domains in the second one, a putative peptidoglycan binding domain (PF01471.17) and cutinase (PF01083.21) suggesting that those ORFs encode Lysin A (LysA) and Lysin B (Lys B), respectively.

The absence of *par*AB genes [9,31] or *int*-*xis* genes [32] in the genome of CRB2 confirms the lytic nature of this phage in spite of the turbid plaques it yields and supports our failure to obtain *M*. *smegmatis* lysogenic cells.

### Comparative genomics of CRB2

**Fig 2:**
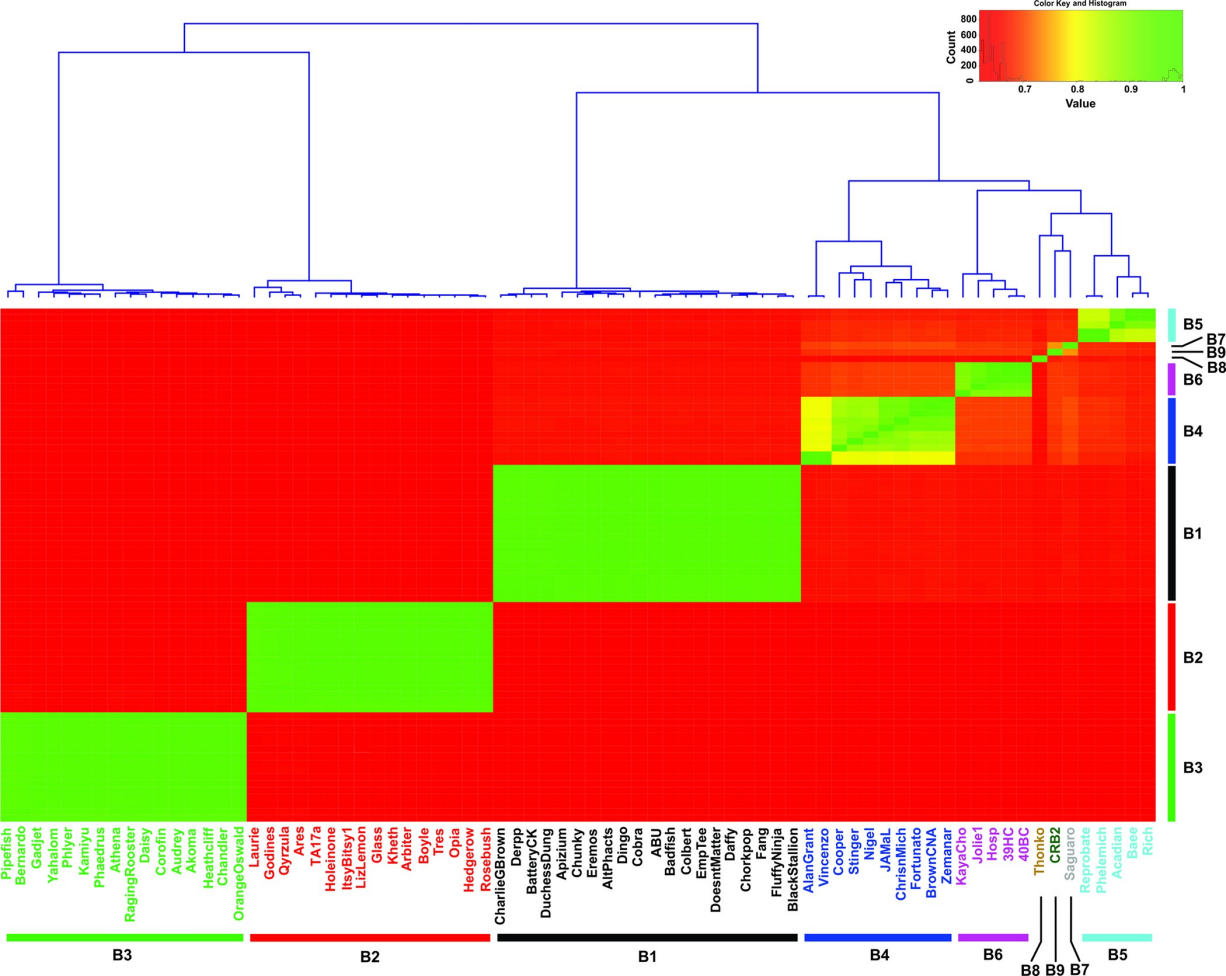
Heatmap representation based on the average nucleotide identity (ANI) distance matrix. Dendrogram at the top showed the hierarchical clustering. Each mycobacteriophage subcluster was represented by different colors.

One of the criteria for mycobacteriophage clustering is the comparison of the average nucleotide identity (ANI) of their genomes [32]. With this objective, a representative group of 75 phages of subclusters B1-B8 (shown in S1 Table) and CRB2 was selected and their ANI values calculated using DNAMaster. This analysis demonstrated that CRB2 remained related to phage Saguaro -a recently described single member defining subcluster B7- with an ANI value of 0.74, but having enough nucleotide differences that prevented both to group together as can be seen in a heatmap representation showing the analysis of ANI (Fig 2).

**Fig 3:**
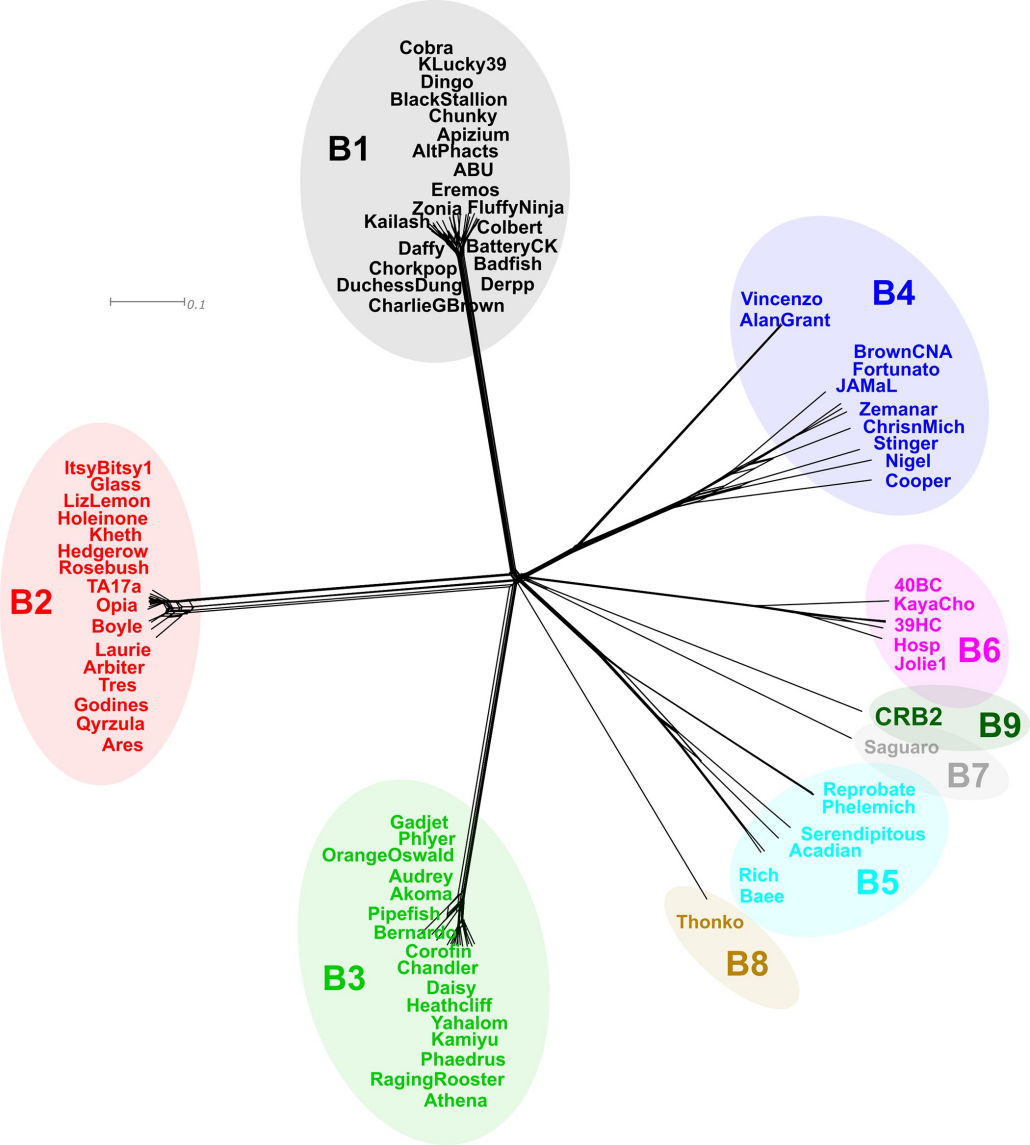
Phylogenetics networks using Neighbor-Net algorithm with equal angle of CRB2 with 75 cluster B mycobacteriophages. Genomes of 75 mycobacteriophages (S1 Table) were compared by Gegenees and the distance matrix generated was analyzed using Splitstree [16]. Colored circles indicate grouping of phages labeled according to their subcluster designations generated by nucleotide sequence comparison. The scale bar indicates 0.1 base substitutions per site.

In order to further analyze phylogenetic distances between CRB2 and the set of mycobacteriophages described above, we applied Gegenees, a program based on fragmented alignment, to compare the mycobacteriophage genomes, generating a distance matrix that was displayed by using NeighborNet analysis in SplitsTree which revealed the phylogenetic distances between each group of phages. As is shown in Fig 3, CRB2 and Saguaro are phylogenetically close but clearly distinguishable, in agreement with the results obtained from the ANI heatmap plot.

**Fig 4:**
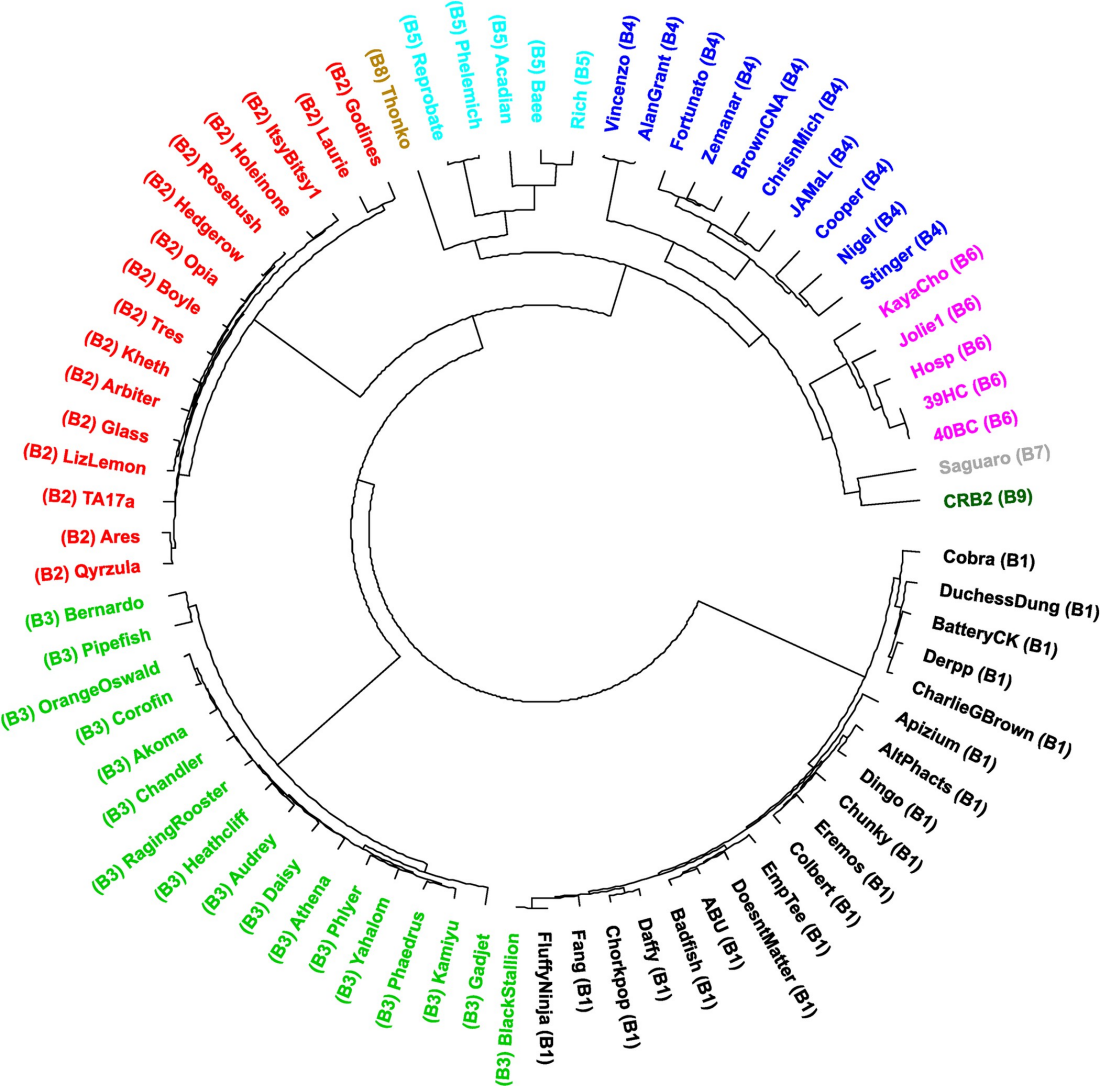
UPGMA dendrogram of 75 mycobacteriophages as listed in S1 Table. Dissimilarity matrix distance was generated using Manhattan analysis (*k*-mer = 8) and CAFE software (25). Dendrogram was constructed using R.

Moreover, we also calculated pairwise dissimilarities among the sequences between a set of B cluster phages using aCcelerated Alignment-FrEe sequence analysis (CAFE) software [25]. Manhattan distances were calculated using a *k*-mer size of 8 (Lu, Y, personal communication). UPGMA clustering was used to construct a dendrogram using this dissimilarity matrix (Fig 4), as expected there is little change on the evolutionary linkage between the B1-B6 subclusters, while phages Saguaro and CRB2 were more related to members of subcluster B6; phage Thonko displays closer relationship to phages belonging into subcluster B5.

A different display form through the use of a heatmap confirmed the higher relation of CRB2 to B6 phages (S1 Fig).

The results obtained were not substantially different than those obtained when using Mash, another method which provides an alignment-free form of comparing genomes [18]; although in this case phage Thonko is located closer to phages Saguaro and CRB2 (data not shown). Intriguingly, the closer relationship of CRB2 is with phages of subcluster B6, all of which excepting for phage KayaCho, were isolated by our lab in Argentina.

**Fig 5:**
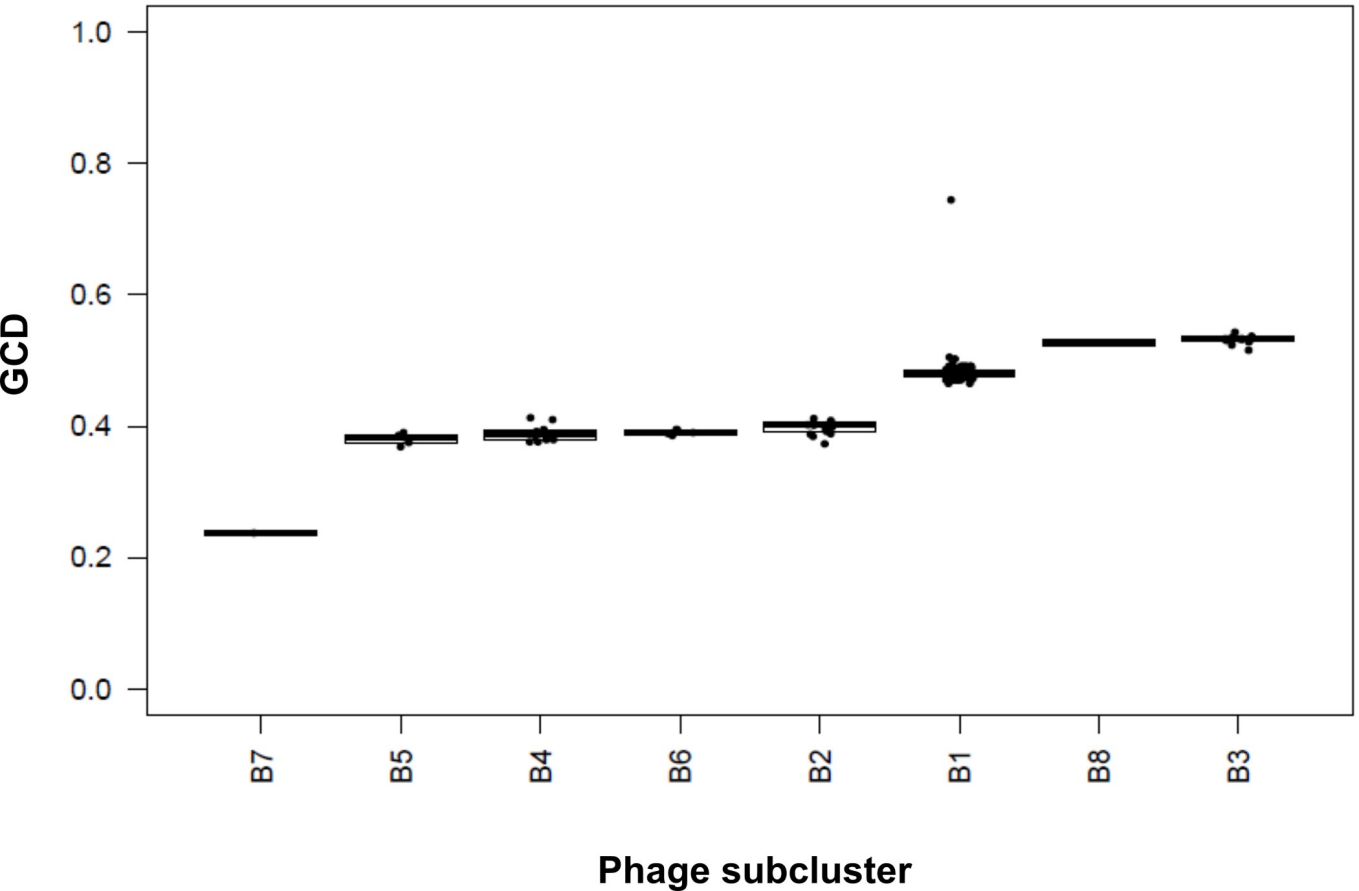
Gene content dissimilarity (GCD). Mycobacteriophage CRB2 was compared with 160 mycobacteriophages (listed in S1 Table) belonging to the different B subclusters. GCD varies between 0 and 1, which correspond to 100% shared genes and no shared genes, respectively.

To further explore the relationship between CRB2 and the rest of the B cluster phages we used a novel measure developed by Hatfull´s group, termed Gene Content Dissimilarity (GCD) [19]. This method has been recently applied to study how Gordonia phages are related to other actinobacteriophages [33]. GCD correlates shared and unshared phams, its values ranging from 1 (no genes are shared) to 0 (identical gene content). For this purpose, we used a set of 160 mycobacteriophage of all the B subclusters. CRB2 showed a close relationship to Saguaro as seen in Fig 5 having a GCD value of 0.24, which means a high number (76%) of shared genes.

Phages from subclusters B5, B4 and B6 displayed a GCD average value of 0.3805, 0.3895 and 0.3909 respectively, a result that could be expected by the ANI and UPGMA clustering (see above). However, B2 phages displayed a comparable GCD value (0.3974), which does not correlate with their relatively low homology to CRB2 as seen in the ANI (Fig 2), heatmap (S1 Fig), and Mash (data not shown) analysis. A possible explanation for this observation is the presence of a block of genes involved in queosine synthesis that is present in B2 phages and may cause a bias in the GCD value. Importantly, phages chosen from subclusters B3 and B8 (Thonko) have the higher values of GCD (0.531 and 0.527, respectively); thus although relatively close to CRB2 (and Saguaro) by both heatmap and Mash, Thonko showed a low percentage of shared phams according to its GCD value (Fig 5).

### Protein phylogeny supports the definition of the new B9 subcluster

#### Comparative analysis of Tape Measure Proteins

**Fig 6:**
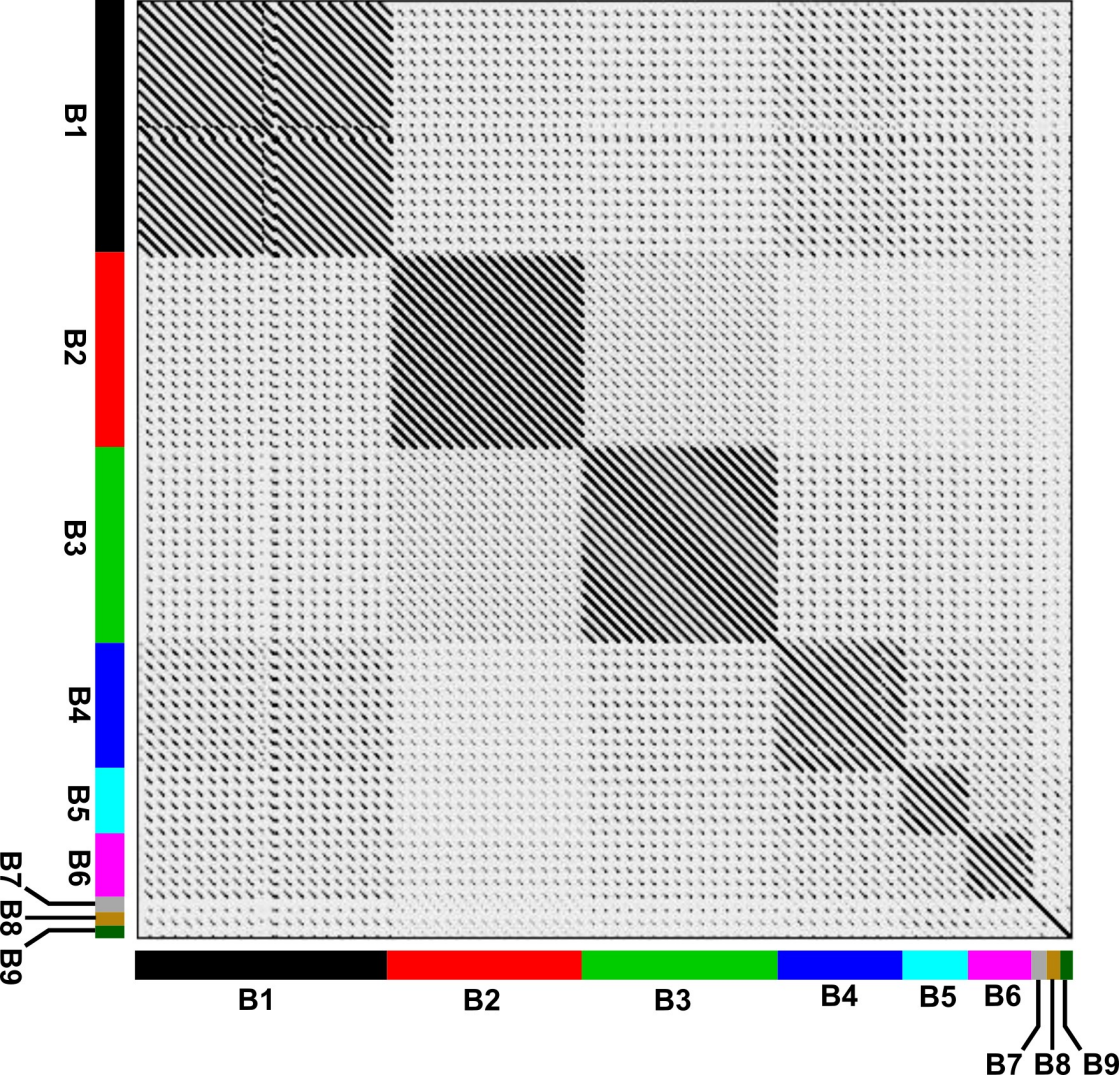
Dotplot comparison of TMPs from 75 mycobacteriophages of cluster B using Gepard. The different subclusters are displayed in colored bars in both axes.Of note, the analysis of the aminoacid sequence of CRB2 TMP revealed a fragment encompassing 69 residues which displayed high homology with a family of proteins known as Resuscitation Promoting Factors (Rpf) as shown in S2 Fig [3]. A comparable domain was also detected in phages Saguaro and Thonko (S2 Fig). Of note, this domain has not been previously described in phages of the B cluster although it has been reported for some phages belonging to different clusters, such as Barnyard and Giles [3,35]. It has been postulated that those proteins are involved in activating or “waking-up” dormant host cells prior infection in the case of phages and in peptidoglycan recycling as a metabolic alarm for quiescent *M*. *tuberculosis* cells and in general for mycobacterial cells. This analysis suggests that CRB2, Thonko and Saguaro may be able to infect *M*. *smegmatis* cells in stationary phase, a hypothesis yet to be tested.

The TMP is a structural protein encoded in almost all phages with non-contractile tails, involved in the determination of the phage tail length [3]. TMP size is directly related to the length of the phage tail. In the case of CRB2 ORF30 (6483 bp) encodes a 2161 AA long TMP, translating into a long tail (estimated ~340 nm as we have previously determined by TEM analysis [9]. Both features are also similar for Saguaro (TMP 6552 bp, tail length ~319 nm), thus making this two phages the ones with the longest tails in the cluster B. TMP has been used to evaluate cluster and subcluster classifications [34], thus we used the amino acid sequence of TMP from the set of 75 phages belonging to different subclusters (B1 to 8) including CRB2 to build a dot plot using Gepard 1.4 (Fig 6). Our results clearly showed that phages Saguaro, Thonko and CRB2 are separated from phages belonging to subclusters B1 to B6, reinforcing the classification of those phages in three different subclusters B7 to B9, respectively.

#### Comparative analysis of the lysis cassette

**Fig 7:**
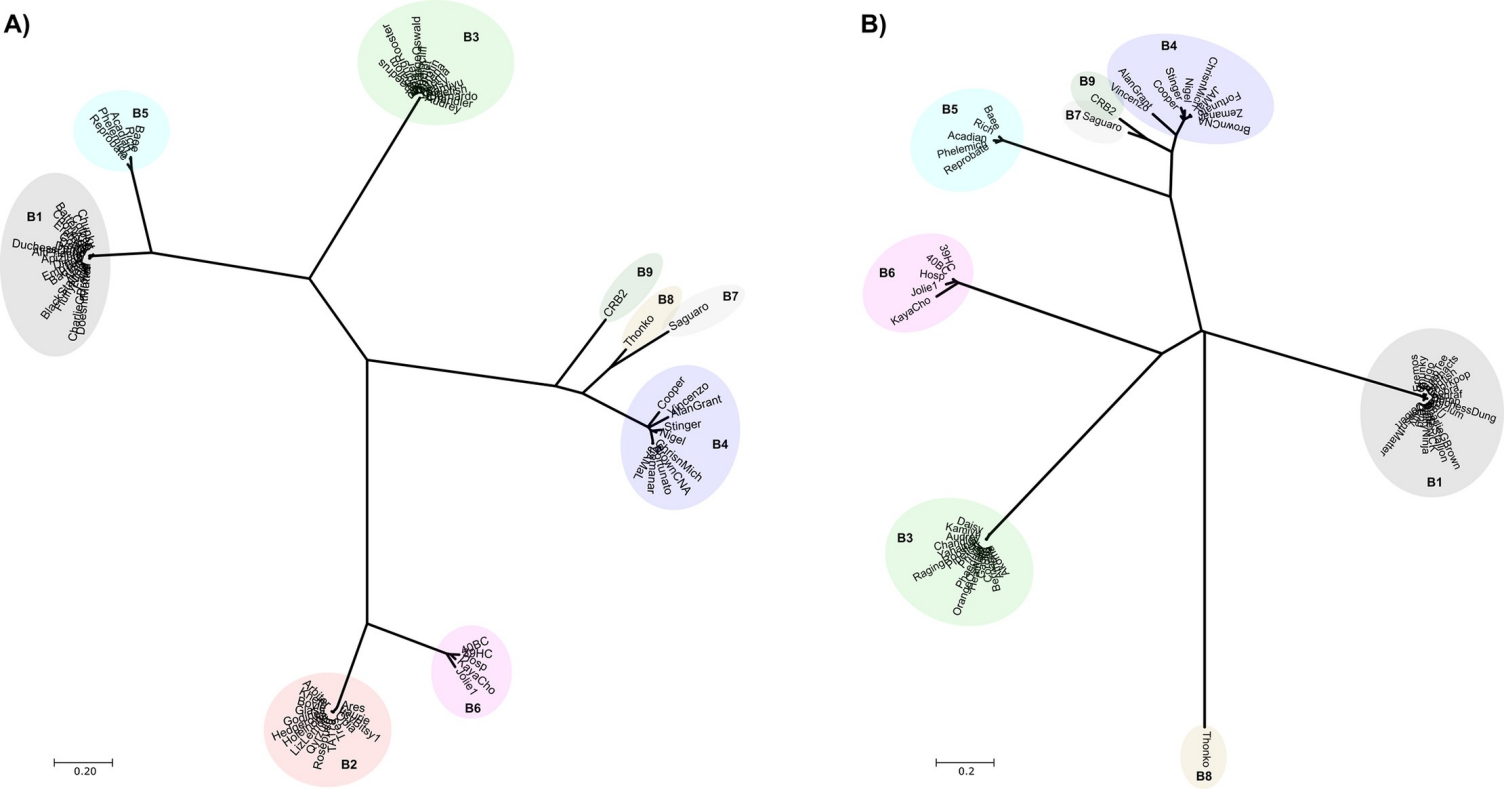
**Neighbor-Joining phylogenetic tree of the amino acid sequences of Lys A (A) and Lys B (B).** Lys A and LysB from a subset of 75 mycobacteriophages (described in S1 Table) were compared. Bootstrap method with 1000 replications was used; the evolutionary distances were computed using the Poisson correction method. All positions containing gaps and missing data were eliminated. The evolutionary distances were computed using the Poisson correction method and are in the units of the number of amino acid substitutions per site. Evolutionary analyses were performed in MEGA7.

The analysis of the lysis cassette of CRB2 indicated the presence of Lys A (gp49) and LysB (gp50) genes but no protein encoding a holin was detected. Phages of the B cluster show different combinations of those gene products, varying from LysA and holin (subcluster B2), LysA and LysB (subclusters B1, B3, B4,B5, B7 and B8) to the unusual disposition observed in the subcluster B6 members in which the genes are in the LysA-holin-LysB order. However, LysA from both Saguaro (subcluster B7) and Thonko (subcluster B8) are highly related, with CRB2 (subcluster B9) related to a lesser degree. The three LysA proteins are less related to B4 LysA (Fig 7A). Nevertheless, while Saguaro and CRB2 have high evolutionary closeness between them and to LysB proteins of phages the B4 subcluster, LysB from Thonko seems to be particularly divergent from all the other LysB proteins under analysis (Fig 7B).

Thus, the analysis of the relatedness of three different proteins (Tapemeasure, LysA and LysB) show that Saguaro and CRB2 are related; however, as shown above, ANI values argue against a common subcluster for both of them.

### GC percentage and genome size

**Fig 8:**
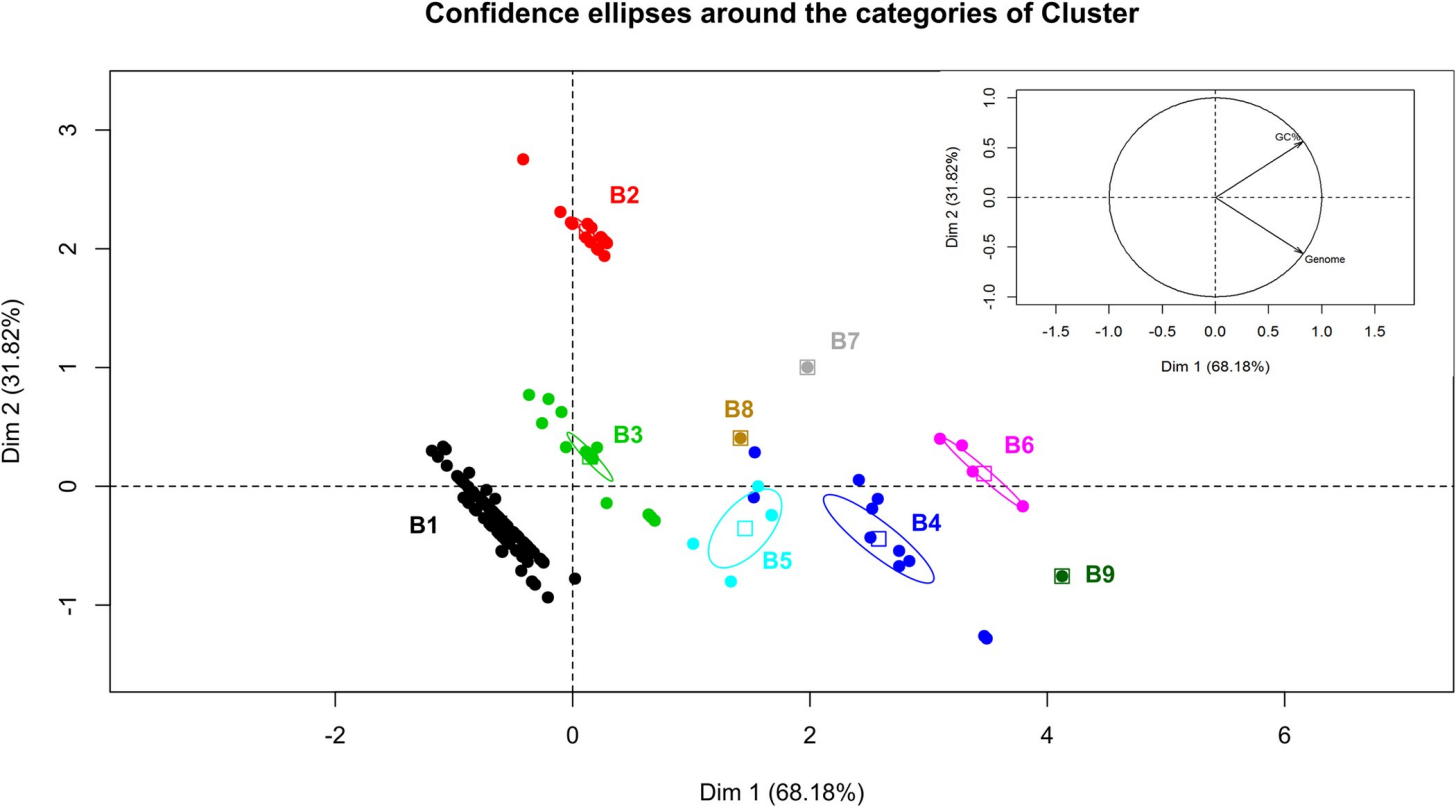
Analysis of the genome size, %GC content and subcluster classification by PCA. This graphics shows the individuals factor map, were each point represent a mycobacteriophage; phages (points) are displayed in colors identifying each B subcluster. Confidence ellipses are plot with α = 0.05. The inset picture represents the variable factor map, where the vectors of genome and %GC content variables are shown.

Mycobacteriophages have a large diversity in genome size ranging from 41 kbp to more than 160 Kpb. Pedulla *et al*. demonstrated a positive correlation between genome size and the %GC content in tailed mycobacteriophages [3], which led us to study if the correlation of genome size and %GC content in the different subclusters. To that end a principal component analysis (PCA) was carried out using FactoMineR package in R. The individuals factor map representation obtained shows the grouping of the phages of each subcluster; confidence ellipses generated for subclusters with more than one member were well separated indicating that each subcluster shared a characteristic genome size and %GC content (Fig 8).

Subclusters such as B1 and B3, included members that shared low values for the variables %GC content and genome size, whereas other subcluster such as B2, is composed of members having high values of %GC content and low values of genome size. The rest of subclusters (B4, B5, B6, B7, B8 and B9) shared high values for both variables, being CRB2 the phage with the largest genome, as shown in Fig 8. Importantly, CRB2 displays a very distinctive position from that of Saguaro and Thonko, again supporting our claim that CRB2 is different enough from Saguaro to define a new subcluster (Fig 8).

### Codon usage preferences as a tool for subcluster organization analysis

**Fig 9:**
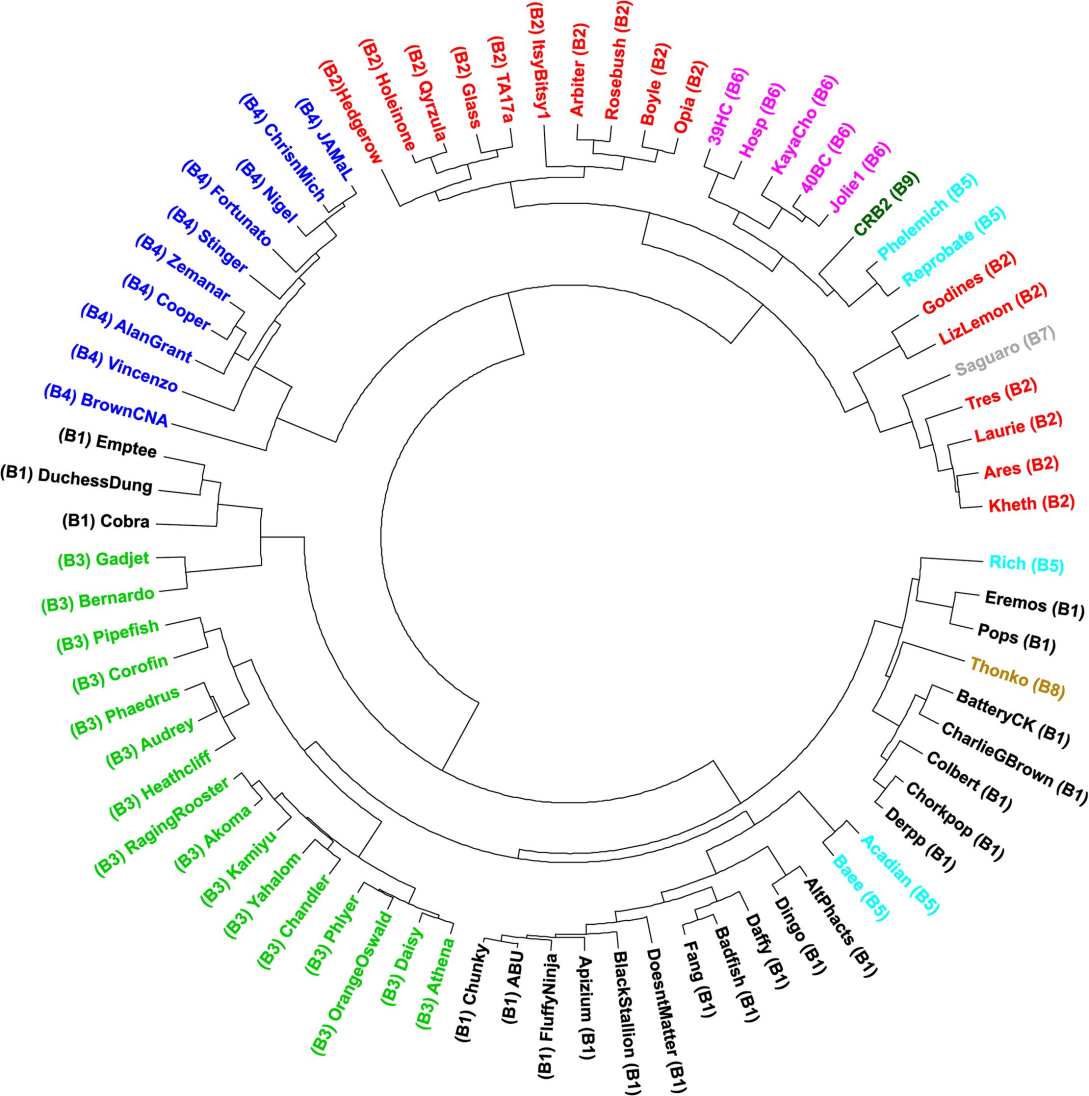
Dendrogram generated by UPGMA clustering using the relative synonymous codon usage (RSCU) values.

To this point, we have shown through different tools that CRB2 merits being in a new subcluster related to phages from subcluster B5, B6 as well as to phage Saguaro (subcluster B7). Interestingly, most of the phages in subcluster B6 were isolated by our laboratory in a region of central Argentina, while the remaining one, mycobacteriophage KayaCho, was isolated in Durban, South Africa). Moreover, in spite of very large numbers of mycobacteriophages isolated and sequenced so far by Hatfull´s group (over 1,600 phages, http://phagesdb.org), mycobacteriophage Thonko (subcluster B8) was also isolated in South Africa and mycobacteriophage Saguaro (subcluster B7) was isolated in Tucson, AZ USA. The relative uniqueness of those mycobacteriophages and the possible similarity of the type of soil where they have been isolated caught our attention and led us to analyze whether that may imply a difference in host preference which may reflect in a difference in codon usage. Esposito *et al*. analyzed the relative synonymous codon usage order (RSCU, which is the ratio of the observed frequency of codons to the frequency expected if all synonymous codons were used equally) for a large set of mycobacteriophages in an attempt to find out if there was a preference for specific mycobacterial species as hosts, but their results did not support that notion [26]. However due to the fact that mycobacteriophages defining the novel B5-9 subclusters were unknown at the time of that publication, we decided to calculate RSCU values to accommodate for those subclusters described more recently. To that end we carried out the analysis on a set of 76 mycobacteriophages randomly chosen from the B cluster (that were previously used for ANI determination, see above); UPGMA clustering produced a dendrogram showing two main branches one of which contained phages from subclusters B2, B4, B6-B9 and some members of subcluster B5 (Fig 9).

Members of subcluster B4 located at a node from which different branches of varying lengths revealed their relatedness in RSCU, however phages from the subcluster B2 arose from a different node and separated in two very different nodes of second and third level, bracketing members of the B6, B7, B8 and CRB2 along with two phages, Phelemich and Reprobate (subcluster B5). Of note, CRB2, B5 and B6 phages were more related at the level of RCSU values, diverging from a common third level node. Interestingly subcluster B5 phages Phelemich and Reprobate were the most similar to CRB2 in RSCU as judged per their position in the dendrogram; however RSCU values of phages belonging into the subcluster B6 phages were also in close correlation with that of CRB2 while Saguaro, which has been consistently close to CRB2 under several bioinformatics tools, was less similar in its RSCU (Fig 9). Of note, all the B6 members (excepting phage Kayacho) as well as CRB2 have a common geographic origin, thus it is intriguing that they share comparable RSCU values which again may suggest a common yet unidentified preferred mycobacterial host. However, as suggested by Esposito *et al*., there is no correlation on RSCU between phages and mycobacterial hosts that may allow establishing a host preference. As a whole our report contributes with the sequence and analysis of a novel mycobacteriophage that joins to the small group of isolates from Argentina which belong to several subclusters containing few members.

## Conclusions

CRB2, a lytic mycobacteriophage isolated in Argentina belongs to mycobacteriophage cluster B, having enough genomic differences to grant it the status of first member of the new subcluster B9. The comprehensive and very thorough work led by G. Hatfull´s team has proposed that mycobacteriophage distribution is better envisioned as a continuum of genetic evolution and thus, the quite often used tools for comparison of genomes and clustering are indeed temporary means to allow researchers to gain a first glimpse to mycobacteriophage evolution. In this regard, it is logical to assume that the more of those biological entities are isolated and sequenced, the closer we will be to have a more realistic perception of their evolution instance at which most probably artificial divisions will fade away. Of note, a substantial majority of the mycobacteriophages isolated so far proceed from USA, with only a handful of isolates coming from South America, mostly from our laboratory. In this regard, in addition to B6, CRB2 and the singleton 19ES–which is very different from any other singleton mycobacteriophage described so far- (Suarez et al, manuscript in preparation), we also have sequenced mycobacteriophages 40AC (falling into a new subcluster, A17), Jolie2 (belonging into subcluster G4 which has few members) and 32HC [8], which along with REM711 falls into subcluster Z (Franceschelli and Suarez, personal communication). Comparison of the locations where mycobacteriophages were isolated in USA, South Africa and Argentina did not reveal geographic or soil characteristics that could give hints on the reason for the relative uncommonness of our findings. Thus, based on the unusually different mycobacteriophages we have isolated from a very small sample size of a few tens of mycobacteriophages, we propose that factors other than the simple relative frequency–perhaps differences in the local, unidentified, host species- are behind the rarity of our phage isolates.

## Supporting information

S1 Table List of mycobacteriophages used throughout this study. Corresponding accession numbers are listed. (^1^) Subset of mycobacteriophages used for ANI heatmap, Splitstree, CAFE, TMP dotplot and RSCU analysis.
(DOCX)

S2 Table List of genes present in mycobacteriophage CRB2. ^a^ Predicted molecular mass of gene product in kilodaltons. ^b^ Function if known or predicted from BLASTP or HHPred analyses. NDM, no database match, other than to other mycobacteriophage proteins. Hyp., database match to a hypothetical protein of unknown function.
(DOCX)

S1 Fig Heatmap visualization of the dissimilarity matrix produced by CAFE. Analysis was performed using the Manhattan distance algorithm based on k-mer counts (with K = 8). The mycobacteriophage subclusters are shown as boxes in the x and y axes. Dissimilarity levels are depicted in colors from blue (low dissimilarity) to light blue (high dissimilarity) as shown in the color bar on the right of the graphic.
(TIF)

S2 Fig TMPs motif analysis. A) Schematic representations of the TMP and resuscitation promotion factor (RPF) domain present in mycobacteriophages of cluster H2 (Barnyard), B7 (Saguaro), B8 (Thonko) and CRB2. B) Alignment of the aminoacid sequence of the RFP domains TMP of mycobacteriophages Saguaro, Thonko, CRB2 and Barnyard, and those present in Mycobacterium tuberculosis proteins containing RpfB and RpfC motifs (Rv_1009 and Rv_1884c, respectively) using ClustalW in MEGA 7. Alignment was edited by Jalview 2.10.5.
(TIF)
