# Supplementary material for: Mycobacteriophage CRB2 defines a new subcluster in mycobacteriophage classification
Source: PLoS One. 2019 Feb 27;14(2):e0212365. doi: 10.1371/journal.pone.0212365 (PMC6392294; doi:10.1371/journal.pone.0212365)
Supplement: S1 Table — Corresponding accession numbers are listed. (1) Subset of mycobacteriophages used for ANI heatmap, Splitstree, CAFE, TMP dotplot and RSCU analysis. (DOCX) [file pone.0212365.s001.docx]

**S1 Table.**

| Bacteriophage | Subcluster | Accession | Bacteriophage | Subcluster | Accession |
| --- | --- | --- | --- | --- | --- |
| ABU^1^ | B1 | JF704091 | LemonSlice | B1 | MF155947 |
| AltPhacts^1^ | B1 | MG962362 | Longacauda | B1 | MF919519 |
| Apizium^1^ | B1 | KR781349 | Lulumae | B1 | MF668276 |
| Ashraf | B1 | KY385380 | Mana | B1 | KX578071.1 |
| Badfish^1^ | B1 | KJ194580 | Manad | B1 | KJ595576 |
| BatteryCK^1^ | B1 | MG962363 | Maskar | B1 | KY385383 |
| BlackStallion^1^ | B1 | KY965066 | Megatron | B1 | MG925348 |
| Chah | B1 | FJ174694 | Mikota | B1 | MF919523 |
| CharlieGBrown^1^ | B1 | KX576647 | MitKao | B1 | KX670813 |
| Chorkpop^1^ | B1 | KY676783 | Morgushi | B1 | JN638753 |
| Chunky^1^ | B1 | MG925339 | Mosaic | B1 | MG925350 |
| Cobra^1^ | B1 | MH051264 | Murdoc | B1 | JN638752 |
| Colbert^1^ | B1 | GQ303259 | Nacho | B1 | JX649098 |
| Daffy^1^ | B1 | KX683293 | Newman | B1 | KC691258 |
| Derpp^1^ | B1 | KX576645 | Numberten | B1 | KJ194583 |
| Dingo^1^ | B1 | MF919503 | Oline | B1 | JN192463 |
| DoesntMatter^1^ | B1 | MG962365 | Olive | B1 | MH077582 |
| DuchessDung^1^ | B1 | MH051251 | OliverWalter | B1 | MG925356 |
| EmpTee^1^ | B1 | KJ567044 | Oosterbaan | B1 | JF704109 |
| Eremos^1^ | B1 | KM236502 | Orion | B1 | DQ398046 |
| Fang^1^ | B1 | GU247133 | OSmaximus | B1 | JN006064 |
| FluffyNinja^1^ | B1 | KP027197 | PG1 | B1 | AF547430 |
| FriarPreacher | B1 | KX576643 | Phamished | B1 | KR816508 |
| Gyarad | B1 | JX649099 | PhatCats2014 | B1 | KX369585 |
| Haimas | B1 | MG770212 | PhenghisKhan | B1 | MG757164 |
| Harvey | B1 | JF937095 | Phergie | B1 | MG757165 |
| Held | B1 | KX683292 | Phipps | B1 | JF704102 |
| Hertubise | B1 | JF937097 | Phunky | B1 | MF919528 |
| Hetaeria | B1 | KT364588 | Piglet | B1 | JX649097 |
| HighStump | B1 | MG757158 | Pinkman | B1 | KX702319 |
| Horchata | B1 | MF919507 | Pipsqueak | B1 | KP027208 |
| ImtiyazSitla | B1 | KY385382 | Pops | B1 | KR997967 |
| Iridoclysis | B1 | KX592589 | Potter | B1 | KU867907 |
| IsaacEli | B1 | JN698990 | ProfessorX | B1 | MG962375 |
| JacAttac | B1 | JN698989 | Puhltonio | B1 | GQ303264 |
| JangoPhett | B1 | MG757159 | Scoot17C | B1 | GU247134 |
| Kailash | B1 | MF919511 | SDcharge11 | B1 | KC661274 |
| Kikipoo | B1 | JN699017 | Serendipity | B1 | JN006063 |
| KingVeVeVe | B1 | KJ538723 | Serpentine | B1 | JX649096 |
| KLucky39 | B1 | JF704099 | Sheila | B1 | MF919530 |
| Lasso | B1 | KM408320 | ShiVal | B1 | KC576784 |
| LeeLot | B1 | MG925346 | Sigman | B1 | KP027209 |
| Bacteriophage | Subcluster | Accession | Bacteriophage | Subcluster | Accession |
| Lego3393 | B1 | KX620786 | Soto | B1 | KJ174157 |
| Squid | B1 | KT599441 | Kamiyu^1^ | B3 | JN699018 |
| Suffolk | B1 | KF713485 | Nozo | B3 | MG925353 |
| Swish | B1 | KJ194579 | OrangeOswald^1^ | B3 | KR080203 |
| TallGrassMM | B1 | JN699010 | Phaedrus^1^ | B3 | EU816589 |
| Thora | B1 | JF957056 | Phlyer^1^ | B3 | FJ641182 |
| ThreeOh3D2 | B1 | JN699009 | Pipefish^1^ | B3 | DQ398049 |
| Trypo | B1 | MG944223 | RagingRooster^1^ | B3 | MG839014 |
| TyrionL | B1 | KX576646 | Yahalom^1^ | B3 | MH051265 |
| UncleHowie | B1 | GQ303266 | AlanGrant^1^ | B4 | KR080200 |
| Virapocalypse | B1 | MF919539 | BrownCNA^1^ | B4 | KT270441 |
| Vista | B1 | JN699008 | ChrisnMich^1^ | B4 | JF704094 |
| Vivaldi | B1 | KM347890 | Cooper^1^ | B4 | DQ398044 |
| Vortex | B1 | JF704103 | Fortunato^1^ | B4 | KX589269 |
| Xavier | B1 | MG944225 | JAMaL^1^ | B4 | KF493881 |
| Yoshand | B1 | JF937109 | Nigel^1^ | B4 | EU770221 |
| Zonia | B1 | KM363597 | Stinger^1^ | B4 | JN699011 |
| Arbiter^1^ | B2 | JN618996 | Vincenzo^1^ | B4 | KR080194 |
| Ares^1^ | B2 | JN699004 | Zemanar^1^ | B4 | JF704104 |
| Boyle^1^ | B2 | MH051249 | Acadian^1^ | B5 | JN699007 |
| Glass^1^ | B2 | KT880194 | Baee^1^ | B5 | KR080199 |
| Godines^1^ | B2 | KR997932 | Phelemich^1^ | B5 | KF416341 |
| Hedgerow^1^ | B2 | JN698991 | Reprobate^1^ | B5 | KF024727 |
| Holeinone^1^ | B2 | MG812490 | Rich^1^ | B5 | KY224000 |
| ItsyBitsy1^1^ | B2 | MG812491 | 39HC^1^ | B6 | KJ433973 |
| Kheth^1^ | B2 | MH001452 | 40BC^1^ | B6 | KJ433975 |
| Laurie^1^ | B2 | KX443696 | Hosp^1^ | B6 | KJ433974 |
| LizLemon^1^ | B2 | KM101117 | Jolie1^1^ | B6 | KJ433976 |
| Opia^1^ | B2 | MG757162 | KayaCho^1^ | B6 | KF024729 |
| Rosebush^1^ | B2 | AY129334 | Saguaro^1^ | B7 | MH744423 |
| Ta17a^1^ | B2 | KF024722 | Thonko^1^ | B8 | MH632120 |
| Tres^1^ | B2 | KT365402 | CRB2^1^ | B9 | MK059749 |
| Qyrzula^1^ | B2 | DQ398048 |  |  |  |
| Akoma1 | B3 | JN699006 |  |  |  |
| Athena^1^ | B3 | JN699003 |  |  |  |
| Audrey^1^ | B3 | KJ194581 |  |  |  |
| Baloo | B3 | MG920059 |  |  |  |
| Bernardo^1^ | B3 | KF493879 |  |  |  |
| ChaChing | B3 | MG925338 |  |  |  |
| Chandler^1^ | B3 | KP027207 |  |  |  |
| Corofin^1^ | B3 | KR080205 |  |  |  |
| Daisy^1^ | B3 | JF704095 |  |  |  |
| Gadjet^1^ | B3 | JN698992 |  |  |  |
| Heathcliff^1^ | B3 | KJ194584 |  |  |  |
