# Supplementary material for: Mycobacteriophage CRB2 defines a new subcluster in mycobacteriophage classification
Source: PLoS One. 2019 Feb 27;14(2):e0212365. doi: 10.1371/journal.pone.0212365 (PMC6392294; doi:10.1371/journal.pone.0212365)
Supplement: S2 Table — a Predicted molecular mass of gene product in kilodaltons. b Function if known or predicted from BLASTP or HHPred analyses. NDM, no database match, other than to other mycobacteriophage proteins. Hyp., database match to a hypothetical protein of unknown function. (DOCX) [file pone.0212365.s002.docx]

**S2 Table.**

| **ORF** | **Putative function** | **Size (AA)** | **Blast Actinobacteriophage database** | **E-value** | **Subcluster** |
| --- | --- | --- | --- | --- | --- |
| **1** | Hypothetical protein (ParB-like) | 210 | Saguaro_Draft_1 | 6e^-89^ | B7 |
| **2** | Queuine tRNA-ribosyltransferase | 329 | Saguaro_Draft_2 | e^-123^ | B7 |
| **3** | QueC-like queosine biosynthesis | 220 | Saguaro_Draft_3 | 2e^-93^ | B7 |
| **4** | QueD-like queosine biosynthesis | 137 | Laurie_4 | 6e^-48^ | B2 |
| **5** | QueE-like queosine biosynthesis | 253 | Saguaro_Draft_5 | e^-107^ | B7 |
| **6** | GTP cyclohydrolase | 188 | Saguaro_Draft_6 | 1e^-88^ | B7 |
| **7** | Terminase small subunit | 185 | KayaCho_3 | 2e^-64^ | B6 |
| **8** | Terminase large subunit | 584 | Saguaro_Draft_8 | 0.0 | B7 |
| **9** | RuvC-like resolvase | 192 | Saguaro_Draft_9 | 2e^-54^ | B7 |
| **10** | Hypothetical protein | 146 | Saguaro_Draft_10 | 1e^-52^ | B7 |
| **11** | Portal protein | 625 | Saguaro_Draft_11 | 0.0 | B7 |
| **12** | Capsid morphogenesis protein | 821 | Saguaro_Draft_12 | 0.0 | B7 |
| **13** | No homology | 45 | Phabba_96 | 8e^-04^ | C2 |
| **14** | Hypothetical protein | 76 | Saguaro_Draft_13 | 4e^-32^ | B7 |
| **15** | Major capsid protein | 616 | Saguaro_Draft_14 | 0.0 | B7 |
| **16** | Hypothetical protein | 265 | Saguaro_Draft_15 | e^-107^ | B7 |
| **17** | Hypothetical protein | 334 | Saguaro_Draft_16 | e^-118^ | B7 |
| **18** | Hypothetical protein | 169 | Cooper_15 | 3e^-33^ | B4 |
| **19** | Hypothetical protein | 151 | Jolie1_13 | 5e^-37^ | B6 |
| **20** | Hypothetical protein | 168 | 40BC_14 | 8e^-55^ | B6 |
| **21** | Hypothetical protein | 110 | KayaCho_15 | 1e^-45^ | B6 |
| **22** | Major tail subunit | 266 | Saguaro_Draft_20 | e^-135^ | B7 |
| **23** | Hypothetical protein | 167 | Saguaro_Draft_22 | 2e^-64^ | B7 |
| **24** | Hypothetical protein | 256 | Saguaro_Draft_23 | e^-100^ | B7 |
| **25** | Hypothetical protein | 173 | Saguaro_Draft_24 | 1e^-71^ | B7 |
| **26** | Hypothetical protein | 109 | Saguaro_Draft_25 | 1e^-35^ | B7 |
| **27** | Tail assembly chaperone | 144 | Saguaro_Draft_26 | 1e^-58^ | B7 |
| **28** | Hypothetical protein | 146 | Saguaro_Draft_54 | 0.012 | B7 |
| **29** | Tail assembly chaperone | 226 | Saguaro_Draft_27 | 7e^-70^ | B7 |
| **30** | Tape measure protein | 2160 | LilMcDreamy_Draft_24 | 0.0 | B |
| **31** | Minor tail subunit | 478 | Saguaro_Draft_29 | 0.0 | B7 |
| **32** | Minor tail protein | 365 | Saguaro_Draft_30 | e^-157^ | B7 |
| **33** | Minor tail protein | 831 | Saguaro_Draft_31 | 0.0 | B7 |
| **34** | Hypothetical protein | 402 | Thonko_30 | 5e^-85^ | B8 |
| **35** | No homology | 153 | Keshu_29 | 3e^-04^ | K3 |
| **36** | Hypothetical protein | 127 | Saguaro_Draft_34 | 2e^-56^ | B7 |
| **37** | Hypothetical protein | 119 | Saguaro_Draft_35 | 5e^-31^ | B7 |
| **38** | Hypothetical protein | 324 | Saguaro_Draft_37 | 3e^-96^ | B7 |
| **39** | Hypothetical protein | 202 | Saguaro_Draft_38 | 2e^-95^ | B7 |
| **40** | Hypothetical protein | 255 | Saguaro_Draft_39 | e^-118^ | B7 |
| **41** | Hypothetical protein | 122 | Jolie1_33 | 7e^-54^ | B6 |
| **42** | Hypothetical protein | 104 | Saguaro_Draft_41 | 4e^-41^ | B7 |
| **43** | Hypothetical protein | 135 | Saguaro_Draft_42 | 7e^-53^ | B7 |
| **44** | Structural protein | 382 | Saguaro_Draft_43 | e^-164^ | B7 |
| **45** | No homology | 77 | Saguaro_Draft_44 | 2e^-07^ | B7 |
| **46** | AlpA-like protein | 68 | Nigel_39 | 9e^-27^ | B4 |
| **47** | HTH DNA binding domain protein | 137 | Schadenfreude_Draft_45 | 2e^-35^ | B1 |
| **48** | Hypothetical protein | 241 | Lephleur_Draft_46 | 2e^-74^ | B2 |
| **49** | Lysin A | 510 | Saguaro_Draft_48 | e^-145^ | B7 |
| **50** | Lysin B | 467 | Saguaro_Draft_49 | 0.0 | B7 |
| **51** | Hypothetical protein | 213 | Saguaro_Draft_50 | 1e^-80^ | B7 |
| **52** | Hypothetical protein | 452 | Nigel_45 | e^-157^ | B4 |
| **53** | Hypothetical protein | 161 | LilMcDreamy_Draft_49 | 6e^-66^ | B |
| **54** | DNA helicase | 595 | Thonko_54 | 0.0 | B8 |
| **55** | Primase/helicase | 916 | Hosp_47 | 0.0 | B6 |
| **56** | Hypothetical protein | 161 | Godines_56 | 1e^-28^ | B2 |
| **57** | DNA pol I | 600 | Saguaro_Draft_57 | 0.0 | B7 |
| **58** | Hypothetical protein | 189 | Nigel_56 | 5e^-43^ | B4 |
| **59** | Hypothetical protein | 134 | Stinger_57 | 8e^-23^ | B4 |
| **60** | Hypothetical protein | 89 | LilMcDreamy_Draft_59 | 9e^-14^ | B |
| **61** | Hypothetical protein | 133 | Acolyte_80 | 2e^-18^ | A2 |
| **62** | Hypothetical protein | 106 | Saguaro_Draft_61 | 5e^-25^ | B7 |
| **63** | Hypothetical protein | 250 | JAMaL_60 | 2e^-60^ | B4 |
| **64** | Hypothetical protein | 80 | Nigel_61 | 4e^-14^ | B4 |
| **65** | Hypothetical protein | 258 | Holeinone_63 | 1e^-37^ | B2 |
| **66** | Hypothetical protein | 77 | Xavier_67 | 2e^-14^ | B1 |
| **67** | Hypothetical protein | 59 | Vincenzo_65 | 2e^-06^ | B4 |
| **68** | Hypothetical protein | 70 | Zaider_Draft_72 | 1e^-11^ | B1 |
| **69** | RNAseE | 236 | Hangman_68 | 6e^-65^ | B4 |
| **70** | Hypothetical protein | 157 | Jolie1_67 | 4e^-31^ | B6 |
| **71** | function unknown | 65 | Low E-value |  |  |
| **72** | function unknown | 90 | Saguaro_Draft_67 | 2e^-15^ | B7 |
| **73** | function unknown | 243 | Thonko_77 | 2e^-67^ | B8 |
| **74** | Hypothetical protein | 183 | JAMaL_71 | 3e^-55^ | B4 |
| **75** | Hypothetical protein | 106 | Thonko_85 | 2e^-38^ | B8 |
| **76** | Hypothetical protein | 54 | No hits found |  |  |
| **77** | Hypothetical protein | 64 | Saguaro_Draft_72 | 1e^-12^ | B7 |
| **78** | HNH endonuclease | 130 | KayaCho_73 | 3e^-50^ | B6 |
| **79** | Hypothetical protein | 66 | Low E-value |  |  |
| **80** | function unknown | 76 | Saguaro_Draft_75 | 1e^-05^ | B7 |
| **81** | Hypothetical protein | 95 | Saguaro_Draft_76 | 2e^-31^ | B7 |
| **82** | Hypothetical protein | 104 | Saguaro_Draft_77 | 3e^-21^ | B7 |
| **83** | Hypothetical protein | 83 | TA17a_81 | 3e^-15^ | B2 |
| **84** | Hypothetical protein | 83 | Jolie1_87 | 7e^-14^ | B6 |
| **85** | Hypothetical protein | 73 | Saguaro_Draft_79 | 3e^-24^ | B7 |
| **86** | Hypothetical protein | 151 | Reprobate_88 | 1e^-49^ | B5 |
| **87** | Hypothetical protein | 346 | Saguaro_Draft_82 | e^-157^ | B7 |
| **88** | function unknown | 89 | Myrna_109 | 2e^-08^ | C2 |
| **89** | function unknown | 50 | Acadian_93 | 5e^-03^ | B5 |
| **90** | function unknown | 137 | Saguaro_Draft_85 | 2e^-35^ | B7 |
| **91** | Hypothetical protein | 125 | Saguaro_Draft_86 | 2e-^18^ | B7 |
| **92** | function unknown | 128 | Low E-value |  |  |
| **93** | function unknown | 91 | Low E-value |  |  |
| **94** | Hypothetical protein | 283 | Saguaro_Draft_91 | 9e^-36^ | B7 |
