# Supplementary figures and images for: Mycobacteriophage CRB2 defines a new subcluster in mycobacteriophage classification
Source: PLoS One. 2019 Feb 27;14(2):e0212365. doi: 10.1371/journal.pone.0212365 (PMC6392294; doi:10.1371/journal.pone.0212365)

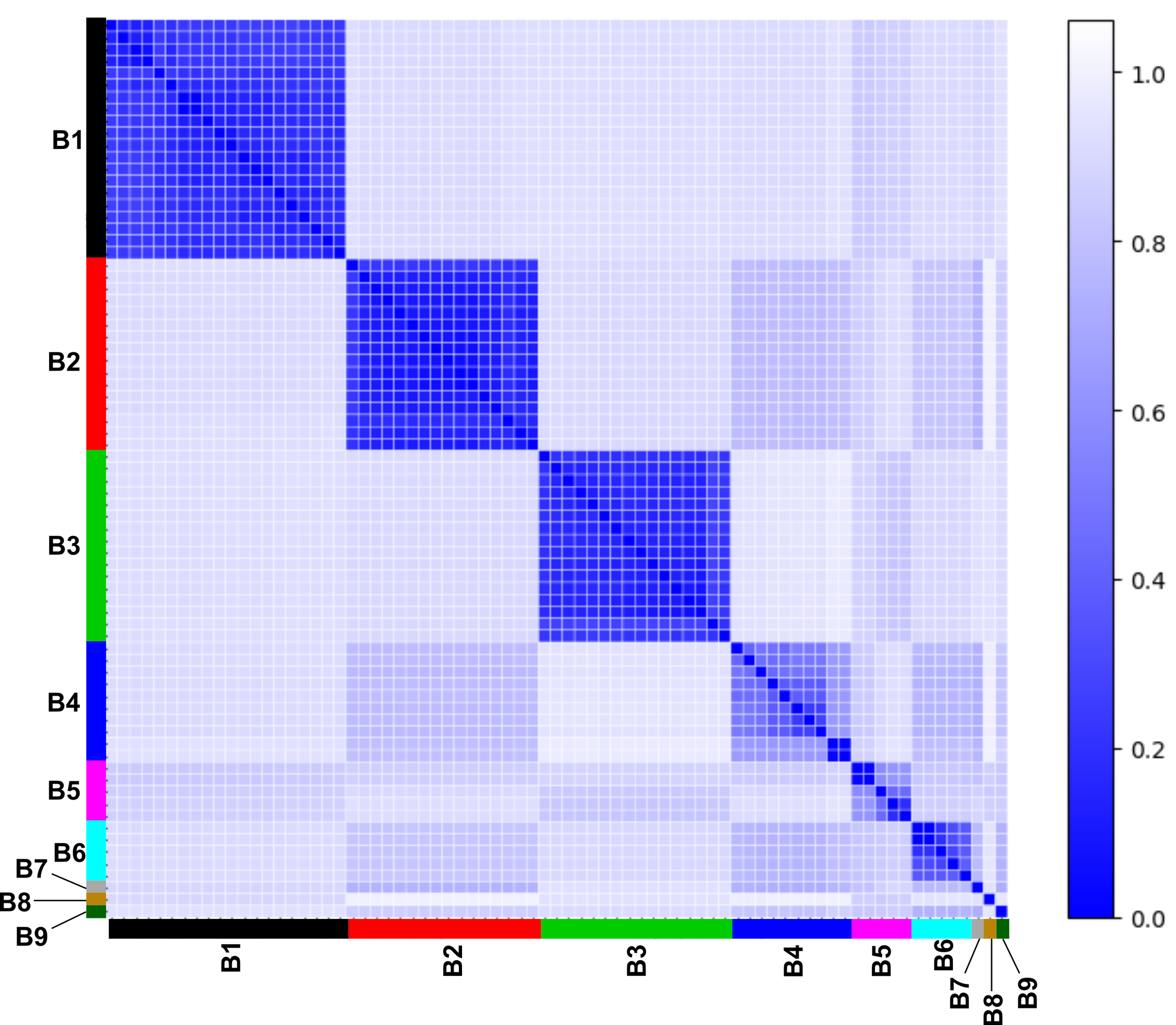

Supplement: S1 Fig — Analysis was performed using the Manhattan distance algorithm based on k-mer counts (with K = 8). The mycobacteriophage subclusters are shown as boxes in the x and y axes. Dissimilarity levels are depicted in colors from blue (low dissimilarity) to light blue (high dissimilarity) as shown in the color bar on the right of the graphic. (TIF) [file pone.0212365.s003.tif]

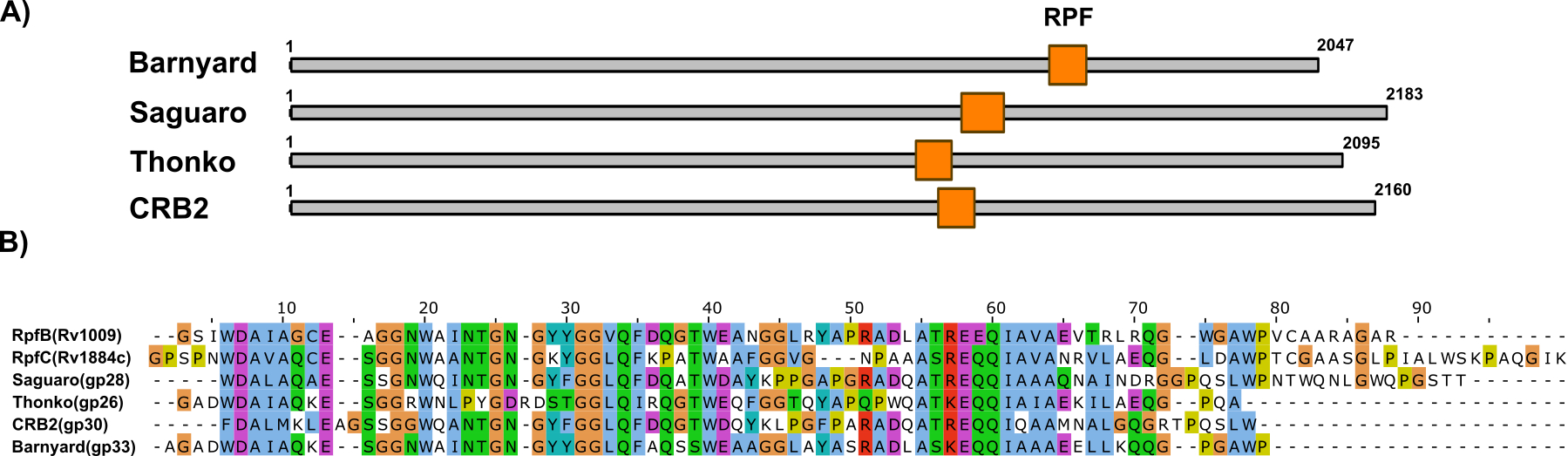

Supplement: S2 Fig — A) Schematic representations of the TMP and resuscitation promotion factor (RPF) domain present in mycobacteriophages of cluster H2 (Barnyard), B7 (Saguaro), B8 (Thonko) and CRB2. B) Alignment of the aminoacid sequence of the RFP domains TMP of mycobacteriophages Saguaro, Thonko, CRB2 and Barnyard, and those present in Mycobacterium tuberculosis proteins containing RpfB and RpfC motifs (Rv_1009 and Rv_1884c, respectively) using ClustalW in MEGA 7. Alignment was edited by Jalview 2.10.5. (TIF) [file pone.0212365.s004.tif]
